# Cultural Responsivity in Technology-Enabled Services: Integrating Culture Into Technology and Service Components

**DOI:** 10.2196/45409

**Published:** 2023-10-03

**Authors:** Elizabeth H Eustis, Jessica LoPresti, Adrian Aguilera, Stephen M Schueller

**Affiliations:** 1 Center for Anxiety and Related Disorders Boston University Boston, MA United States; 2 Department of Psychology Suffolk University Boston, MA United States; 3 School of Social Welfare University of California Berkeley Berkeley, CA United States; 4 Department of Psychiatry and Behavioral Sciences University of California San Francisco San Francisco, CA United States; 5 Department of Psychological Science University of California Irvine Irvine, CA United States

**Keywords:** technology, mobile health, mHealth, mental health, cultural responsivity, human support, mobile phone

## Abstract

Technology-enabled services (TESs) are clinical interventions that combine technological and human components to provide health services. TESs for mental health are efficacious in the treatment of anxiety and depression and are currently being offered as frontline treatments around the world. It is hoped that these interventions will be able to reach diverse populations across a range of identities and ultimately decrease disparities in mental health treatment. However, this hope is largely unrealized. TESs include both technology and human service components, and we argue that cultural responsivity must be considered in each of these components to help address existing treatment disparities. To date, there is limited guidance on how to consider cultural responsivity within these interventions, including specific targets for the development, tailoring, or design of the technologies and services within TESs. In response, we propose a framework that provides specific recommendations for targets based on existing models, both at the technological component level (informed by the Behavioral Intervention Technology Model) and the human support level (informed by the Efficiency Model of Support). We hope that integrating culturally responsive considerations into these existing models will facilitate increased attention to cultural responsivity within TESs to ensure they are ethical and responsive for everyone.

## Introduction

Technology-enabled services (TESs) are clinical interventions that combine technological and human components to provide effective and efficient health services [1]. A large body of the literature has demonstrated the efficacy of TESs in mental health, especially for anxiety and depression in adults [2-5]. As such, TESs for mental health are recommended and used as frontline treatments around the world [4,6,7]. TESs have the potential to significantly increase the reach of evidence-based treatments and address multiple barriers to accessing care if designed and implemented to address the needs of diverse communities. As such, work is being conducted to understand how to effectively integrate TESs into clinical workflows [8-10] and how to prepare the clinical workforce to provide support through TESs [11,12].

**Figure 1:**
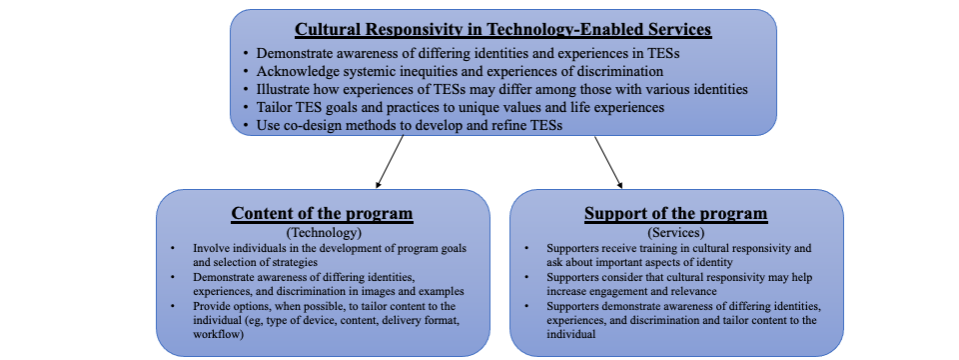
Cultural responsivity in technology-enabled services (TESs).

It is hoped that these interventions will be able to reach diverse populations across a wide range of identities (eg, race, ethnicity, sexual orientation, gender, and social class), who otherwise may not have access to quality and effective care [13,14]. In this regard, TESs have the potential to expand access and decrease disparities in mental health treatment. To date, this potential has largely been unmet. However, more recently, studies have attempted to realize this hope by adapting, tailoring, or co-designing TESs for specific populations or identities [15-22]. Yet, the field has lacked overarching guidance on how to consider cultural responsivity in these interventions and what the targets for such adaptations, tailoring, or design should be. The purpose of this paper is to propose a framework for integrating the consideration of cultural responsivity into existing models of technology development and support services and to highlight the need for cultural responsivity within all TESs, including those developed for the general population. Aligned with the definition of a TES as comprising technology and human service components, we argue that cultural responsivity needs to be considered in each of these components (Figure 1). First, we discuss the need to address cultural responsivity in TESs. Second, we provide considerations related to cultural responsivity with regard to the content of TESs. Finally, we focus on cultural responsivity in the provision of human support in TESs and offer directions to move the field forward.

## The Need to Address Cultural Responsivity in TESs

Significant, long-standing disparities exist in access to traditional face-to-face mental health care for marginalized and minoritized groups. For example, significant disparities have been documented between White people and people who are racially and ethnically minoritized in the United States [13,23-26]. The sources of these long-standing disparities in access to quality and effective care stem from racism, a history of mistreatment of people who are racially and ethnically minoritized in medical settings, experiences of discrimination, and a lack of culturally responsive mental health care [27]. In addition, individuals from ethnic minority groups; those who identify as gay, lesbian, bisexual, or transgender; and those who report lower social class are also less likely to access mental health treatment, yet little research has addressed or examined barriers to treatment for various marginalized aspects and intersections of identity [14,28].

Despite the promise of digital interventions to increase access, reach diverse populations, and reduce mental health treatment disparities, most reported consumers of TESs are not representative of the diversity of the entire population. Only a small number of studies in the literature report on TES use based on demographic variables, and those that do tend to only report on limited variables, most commonly age and gender [29]. Some evidence suggests that there may be differences in who accesses and uses TESs, given that digital interventions are most frequently used by middle-aged White women, who are also the most frequent consumers of in-person therapy services [18]. Furthermore, among individuals who used a TES for chronic disease management in the United States, those who identified racially as White had significantly higher use of tools within interventions than those who identified as people who are racially minoritized [29]. Another study found significant usability barriers in commercially available apps for mental and physical health concerns in an older and low-income sample that was racially diverse [30]. Furthermore, Aguilera and Berridge [31] found differences in how Spanish- and English-speaking participants perceived a texting intervention. Qualitative data indicated that Spanish-speaking participants described the most helpful aspect of the intervention as feeling cared for and supported, whereas English-speaking participants described the most helpful aspect as increased self-awareness of mood. Collectively, these findings suggest that marginalized and minoritized groups may not be accessing TESs or using them in the same manner as other groups. We argue that a lack of cultural responsivity within TESs is likely to be at least partially responsible for such differences. Individuals from marginalized and minoritized groups may feel, appropriately so, that TESs are not built for them and may find that they do not feel relevant. For example, mainstream TESs may include a focus on values such as individualism that may not resonate for individuals from cultures that tend to be more collectivist or that place a higher value on family and community. In addition, these approaches generally do not discuss experiences of racism or discrimination and may not have representation across identities in examples or vignettes. This is often the case because individuals from marginalized and minoritized groups have not been adequately incorporated into the development process. This literature also indicates that the treatment disparities observed in face-to-face care persist with digital interventions. Thus, digitizing interventions alone may not overcome mental health disparities and might actually increase them.

Given these documented disparities, developing TESs in a culturally responsive manner is clearly needed to increase access and relevance. Although the broader literature has had limited consideration of cultural responsivity [32], a recent systematic review found the perceived fit of a TES to be a facilitator of engagement [33]. Perceived fit in this review included whether the intervention seemed relevant to the individual, their culture, and values and whether there were options for personalization versus assuming that one size fits all. These results highlight the importance of considering culture in TESs. In this paper, we propose a general framework for designing culturally responsive TESs that can be applied across a wide range of domains of identity, including but not limited to race, ethnicity, gender, sexual orientation, social class, ability, religion and spirituality, and immigration status. Within the context of the United States, certain identities within these domains have been marginalized or minoritized. For example, within the United States, marginalized and minoritized groups include people who are racially and ethnically minoritized; people who identify as transgender, nonbinary, gay, lesbian, bisexual, or queer; and those with lower social class, among others. We recognize that many marginalized and minoritized identities exist in the United States and worldwide. This list is intended to provide several examples, and we acknowledge that many identities are not represented here. Individuals with marginalized and minoritized identities often experience discrimination and barriers to quality and effective health care. Therefore, we believe that cultural responsivity should include a wide range of identities and consider intersectionality, which examines how various aspects of identity such as race, sexual orientation, and gender interact [34,35].

## Cultural Responsivity

Given the need to provide effective care for diverse populations, the next step is to identify best practices for integrating the understanding of cultural differences into treatment and adapting care accordingly. As defined in traditional treatments, cultural responsivity involves the therapist (1) demonstrating awareness of the significance of differing identities in the context of therapy, (2) acquiring and demonstrating knowledge about the modal experience of clients with specific identities, and (3) tailoring therapy goals and practices to the unique values and life experiences of each individual client [36]. Cultural responsivity is an important aspect of the therapeutic relationship with every client, regardless of whether the client and therapist share identities.

Cultural responsivity is different from cultural competence, which is defined as “a set of congruent behaviors, attitudes, and policies that come together in a system, agency, or among professionals that enables effective work in cross-cultural situations” [37]. Cultural competence tends to generalize the experience of individuals within any one group and ignore the unique experiences and presentation of individual clients within their specific cultural context [38]. Furthermore, this approach tends to reify stereotypes and, within racial and ethnic groups, can perpetuate cultural racism [39].

Cultural humility is another approach that moves beyond competency and focuses on the interactions between the therapist and client. Cultural humility includes “(a) a lifelong motivation to learn from others, (b) critical self-examination of cultural awareness, (c) interpersonal respect, (d) developing mutual partnerships that address power imbalances, and (e) an other-oriented stance open to new cultural information” [40,41]. Although we use cultural responsivity, owing to its active approach, as the main term in this paper, there is significant overlap between cultural responsivity and cultural humility.

To date, most research has focused on cultural responsivity when services are delivered face to face [35,42-44]. However, the US Department of Defense has developed practice guidelines for integrating technology into clinical care that include a focus on cultural considerations [45]. Additional research is urgently needed to address the disparities already seen with TESs and to ensure that TESs do not increase treatment disparities and that they are responsive to diverse individuals’ needs and contexts. Otherwise, these programs may not be helpful and may even be harmful. Although cultural responsivity may look different in the context of a TES compared with an intervention delivered face to face, each aspect of the definition of cultural responsivity introduced earlier—(1) demonstrating awareness of identities, (2) demonstrating knowledge of different experiences, and (3) tailoring to the individual (Textbox 1)—can be addressed both in terms of the content of programs and human support.

**Components of cultural responsivity to consider in technology-enabled services informed by the work of Jones-Smith [36].**

1. Demonstrating awareness of the significance of differing identities
2. Acquiring and demonstrating knowledge about different experiences related to identities
3. Tailoring goals and practices to the unique values and life experiences of the individual

## Program Content

### Overview

In terms of program content, TESs are either typically developed for a specific population, in which case content is tailored to that population, or delivered broadly and therefore are not tailored to any specific population (ie, developed for adults with anxiety and depression). When interventions are not intentionally tailored in the United States, the default assumption is typically that the target consumers are White middle-class individuals, potentially worsening the existing disparities. These unconscious assumptions about who the target consumer is are often because most developers of these programs also match this demographic description and draw on their own experiences to generate content. Given the inherent focus on specific identities and intersectionality in tailored interventions, some aspects of the development process may differ for tailored versus nontailored programs. Here, we focus on broad recommendations that could be used across a range of programs (ie, both tailored and nontailored) to consider targets for cultural responsivity.

### The Behavioral Intervention Technology Model

The Behavioral Intervention Technology (BIT) Model is an existing, comprehensive framework that can be used to inform the development of TESs and considers conceptual, technical, and clinical aspects of an intervention [46]. It is unique in that it includes both behavioral principles and technological features. For example, it includes theoretical considerations, such as the clinical aims of the intervention (*why* an intervention is developed and what the goal is), and behavior change strategies (*how* the aims are achieved). It also includes considerations at the level of instantiation, such as the elements (the *what*, eg, information delivery, notifications, and logs), the technical characteristics (the *how* the intervention works technically, eg, esthetics and medium), and the workflow (the *when*, eg, user defined, frequency, and tunneling). Therefore, the BIT Model can be used to consider various targets for adaptations, tailoring, and design related to cultural responsivity for content in TESs.

### Recommendations for Developing Content of the Technology

#### Overview

One important consideration is the process of content development and who is involved. Given the documented concerns about the usability and use of TESs by diverse populations [29,30], experts have recommended the use of participatory methods or a co-design process that includes diverse stakeholders in the development process to support representation and tailoring [47,48]. Therefore, a co-design process should be used across all aspects of the BIT Model. In addition, different parts of the BIT Model can be used to address each part of the definition of cultural responsivity (Textbox 1).

#### Why: Clinical Aims of Content

Co-design is helpful at all stages of development and is especially important at the beginning of the process when identifying *why* an intervention should be developed and what the goals of the intervention are. The intended audience should be included in the discussion on defining the problem, identifying the current resources available, why an intervention is needed, and what the goals should be. These decisions will have a significant impact on other aspects of the intervention downstream (eg, which behavior change strategies are included). For example, individuals may have different perceptions of what the *problem* is and what is needed to address it compared with the developers of a TES, and therefore their goals may vary (eg, a focus on reducing symptoms vs increasing wellness or increasing social support). Program developers could identify a type of technology (eg, an app) and a framework as a starting point (eg, a cognitive behavioral therapy [CBT] framework). Developers will then need to work with the target audience to make decisions about the technology and conceptual framework by considering the culture and context of the target audience.

#### How: Behavior Change Strategies

The existing literature on TESs indicates that cultural preferences might impact the type of content that individuals with various identities may prefer or find helpful. For example, Aguilera and Berridge [31] examined qualitative data from an SMS text messaging intervention and found that Latinx Spanish speakers reported that feeling supported by the messages (ie, social support) was most helpful, whereas English speakers (mostly White and African American) reported increased introspection and self-awareness of their mood as being most helpful. These findings indicate that people perceive interventions based on what they value, and a cultural responsiveness approach can consider and integrate these perspectives. McCall [49] reported that African American women in one sample indicated that they would like specific types of information (eg, information about Black female therapists in their area) and inspirational messages to be included in the app content for managing anxiety and depression. These preferences could be used to inform how an intervention attempts to achieve its goals and what type of content or strategies are included.

Once specific behavior change strategies are selected, the content should include an awareness of differing identities, the demonstration of the knowledge of systems of privilege and marginalization, and experiences of discrimination [42,50,51]. For example, when introducing cognitive restructuring, instructions should clearly state that thoughts related to the validity of experiences of discrimination should not be questioned or restructured. For example, thoughts such as “My boss is going to say something racist to me at work” should not be restructured. However, thoughts related to the internalization of discrimination (eg, “I don’t belong here”; “I am bad at my job”; “I am not valuable or worthy”; or “People will never value me”) may be helpful to target [42,50]. Furthermore, when introducing psychoeducation and behavior change skills, the adaptive function of avoidance in certain contexts should be acknowledged and that helpful behaviors can vary across contexts. Decisions about behavior change can also be connected to individuals’ goals and values to tailor content to the individual. In addition, content on pleasant activity scheduling should include a range of example activities that are free or low cost, are accessible, and involve being in a community or with family in addition to more individualistic activities. Content on emotions should consider cultural differences in the experience, interpretation, regulation, and expression of emotions, and content related to interpersonal relationships should consider differences in interpersonal functioning and cultural norms.

#### What and How: Elements and Technical Characteristics

It is important to consider that individuals may have preferences for different types of notifications, passive data collection, and tracking logs. Preferences also relate to the delivery format of content and ways to use culturally relevant delivery approaches. For instance, some interventions and health education tools have been delivered in telenovela or fotonovela format [52,53]. One example of this is a culturally adapted depression education intervention delivered via a fotonovela in a comic book style that included photographs, captions, and soap opera narratives about a Latina woman’s experience with depression [52,53]. Furthermore, African American women in one sample recommended including a group chat feature in a culturally tailored app so that consumers could interact with each other [49]. Program esthetics, such as photos and images, names, and examples, should reflect a range of identities and experiences and be mindful of intersectionality. Certain elements and technical characteristics may be selected for tailored programs, whereas options may be provided to individuals when possible in both tailored and nontailored programs. Innovative work has begun to use methods such as machine learning algorithms to tailor TESs to individuals, including individuals from low-income ethnic minority groups [54].

#### When: Workflow

Furthermore, differences exist in device availability and preference, as well as in the ability to use certain device features. Research indicates that people who identify as African American and Latinx are more likely to use smartphones to access the internet than other devices [55], and 1 study found that a sample of African American women had concerns about SMS text messaging as a modality related to concerns about privacy, confidentiality, and messages feeling impersonal [16]. Consumers might also differ in their preferences regarding when content is available or released (eg, user defined vs tunneled). When feasible, it is important to assess workflow preferences to inform the development of a program or to provide different options (eg, ability to access the program on various devices and option to receive content via SMS text messages or another format).

Certainly, TESs range in content, length, and delivery format (eg, 1 session to ≥8 sessions and apps vs web-based programs), so what culturally responsive content looks like will vary across programs. In addition to the available research on TESs, guidelines on the provision of culturally responsive, evidence-based, face-to-face interventions should be incorporated into the development of content for TESs [35,42,44,56]. We offer some considerations for culturally responsive content in Table 1. One limitation in terms of content is that it will likely never be possible to include examples that every single individual can relate to or to provide options for every preference an individual may have. Developers should ensure there are a range of examples in general programs, even if they do not represent everyone, and that options are available when feasible; this may still be helpful and demonstrate an awareness of differing identities and experiences and an attempt to tailor content.

If content is culturally responsive, it may help prevent potential harm individuals would otherwise experience when interacting with programs and individuals may feel more represented in or validated by the content. In addition, culturally responsive content may provide a helpful foundation for the human support component of TESs. For example, supporters (ie, the people providing the human support component) can refer to culturally responsive content when questions or concerns from consumers arise later. However, although it is important that content is culturally responsive, once a program is developed, the content is often static, highlighting the need to also focus on the dynamic role that support can play in cultural responsivity.

**Table 1:** Examples of culturally responsive program content.

Level of BIT^a^ Model and topic				Recommendation for potential content		Aspects of cultural responsivity^b^
**Theoretical**						
	**Why: clinical aims**					
		Who is creating the aims of the intervention and what are their goals and values?	Use co-design methods to involve others (ideally target audience) in the process of determining why a TES^c^ is needed, how it could be helpful, what the aims are, and what the goals should be. For example, goals may vary from reducing symptoms to increasing wellness or increasing social support.		(1) and (2)	
	**How: behavior change strategies**					
		Preferences about type of content	Individuals may find different types of content more or less helpful. Some people may prefer supportive content or messages and others may prefer skill-based content. If possible, allow individuals to select content based on preferences. In addition, machine learning algorithms can be used to tailor content.		(3)	
		Psychoeducation about privilege, marginalization, and discrimination	Acknowledge systems of privilege and marginalization. Acknowledge that individuals may experience discrimination based on a range of identities. Acknowledge the adaptive nature of avoidance in response to threat.		(1) and (2)	
		Psychoeducation on emotions	Consider that although emotions are broadly universal, the experience, interpretation, regulation, and expression of emotion varies across cultures.		(1) and (2)	
		Cognitive restructuring	Instructions should make clear that thoughts related to the validity of experiences of discrimination should not be questioned or restructured. Thoughts related to the internalization of discrimination may be helpful to target.		(1) and (2)	
		Behavior change	Discuss the adaptive function of avoidance in certain contexts, connect decisions to individuals’ goals and values, and highlight that helpful behaviors can vary depending on the context. Ensure examples for pleasant activities include free and low-cost accessible options, along with activities that include community and family in addition to more individualistic activities.		(1) and (2)	
		Interpersonal functioning	Content about interpersonal functioning and relationships should acknowledge differences in relational styles, cultural norms, and preferences and help individuals determine what types of interpersonal interactions are helpful for them.		(1), (2), and (3)	
		Throughout program	Vignettes and examples should include experiences of discrimination with diverse minoritized populations, a range of pronouns, lesbian, gay, bisexual, and queer romantic relationships, and activities such as going to a church, temple, mosque, etc.		(1) and (2)	
**Instantiation**						
	**What and how:** **elements and technical characteristics**					
		Preferences for types of notifications, tracking, and messaging	Use co-design methods to involve those you are hoping to reach to understand preferences. If developing a general program, consider that there may be differences in preferences and ability and include various options when possible (eg, notifications, button size, navigation, instructions, passive data collection, and different types of tracking logs).		(3)	
		Medium	Consider different delivery formats and features that are familiar and relevant (eg, telenovela and group chat feature).		(3)	
		Program esthetics	Photos, names, narration, and examples should reflect a range of identities, and be mindful of intersectionality.		(1)	
	**When:** **workflow**					
		Device preferences and availability	Consider preferences for the type of technology (eg, SMS text messages vs computer vs app) and device availability and design accordingly. Consider programs that can be used across different devices.		(3)	
		Consider different preferences for workflow	Use co-design methods, consider allowing individuals to make decisions about the timing of the program and access to content (eg, user defined, tunneled, or event based).		(3)	

^a^BIT: Behavioral Intervention Technology.

^b^(1) Demonstrating awareness of the significance of differing identities; (2) acquiring and demonstrating knowledge about different experiences related to identities; and (3) tailoring goals and practices to the unique values and life experiences of the individual [36].

^c^TES: technology-enabled service.

## Support

### Overview

The provision of human support is another pathway through which TESs might become culturally responsive. One consistent finding is that TESs that include some form of support yield significantly larger effect sizes and significantly lower dropout rates than interventions without support [57-60]. However, most research has focused on the presence or absence of support, with only some research examining the quantity and content of support [61,62] and the training or background of the supporter [60,63]. Important questions remain regarding *how* and *why* support is helpful with regard to engagement and outcomes.

Support may be especially important when working with individuals with marginalized and minoritized identities [64]. Furthermore, targeting support may have additional benefits because it is dynamic and often provided across the course of a TES versus content within the technology itself, which once developed, tends to be more static. For example, support can address questions or concerns that arise with content and skill application, which may otherwise be left unaddressed. However, if support is not culturally responsive, negative consequences might occur. As individuals complete program content and practice assignments, they may have questions about how to apply certain skills, given certain aspects of their identity or context or experiences of discrimination. If supporters are unable to respond to these questions and concerns in a culturally responsive way, individuals may, understandably, stop using the program, not benefit from the program, and may not want to engage with mental health treatment in the future.

The medium (eg, SMS text messages, phone calls, and asynchronous written messages) and frequency (eg, weekly or as needed) of support vary, as does the type of support in existing TESs. Some TESs include technical support (eg, addressing issues with technology or the program), others provide support focused on increasing engagement (eg, supportive accountability [65]), and some provide clinical support (eg, the Macquarie University Model [66] and the Swedish Experience [67]). Some research indicates that support can be delivered by people with minimal professional training, which can afford benefits in terms of cost-effectiveness, scalability, and the sustainability of these programs [60,63,68]. Peers can also deliver support [69]. Despite differences in support protocols, many are similar in that in most supported TESs, consumers are assigned a supporter or coach who reviews their engagement with the program and practice assignments often at set intervals, provides some type of encouragement or feedback, and is available to answer questions.

### The Efficiency Model of Support

Schueller et al [70] developed the Efficiency Model of Support, which includes a theoretical model of 5 treatment failures that frequently arise in TESs and posits that to be more effective, support interactions should target these treatment failures. This model advances the field in that it begins to examine common problems (“failures”) that arise in TESs and to move beyond examining the presence or absence of support to investigate the function of support. The failures identified in this model are usability, engagement, fit, knowledge, and implementation. A *usability* failure refers to barriers or problems with the intervention or the technology itself. An *engagement* failure occurs when someone has the ability to access the intervention but does not, perhaps because of low motivation. A *fit* failure is when the assigned content does not match an individual’s needs or symptoms. A *knowledge* failure is when an individual uses a tool but not in the correct manner. Finally, an *implementation* failure occurs when an individual is able to use a skill correctly within a TES but does not apply the skill in their daily life. Usability failures occur at the level of the program, whereas the remaining 4 failures focus on use, content, and skill application and are more relevant for interactions between the supporter and individual or the translation of knowledge contained from the technology into one’s life. Multiple failures can occur simultaneously, and one type of failure (eg, fit) can lead to another failure (eg, engagement).

### Recommendations for the Provision of Support

#### Overview

We suggest that cultural responsivity is a lens through which one could consider these 5 potential failures, consistent with the Efficiency Model of Support. In other words, a lack of cultural responsivity at the levels of the program and support could contribute to all of these failures. Considering these failures through the lens of cultural responsivity could help supporters identify and respond to these common problems (Multimedia Appendix 1). This lens could facilitate the provision of culturally responsive support that addresses each aspect of the definition of cultural responsiveness (Textbox 1). It is important to note that failures related to a lack of cultural responsivity reflect a failure or limitation of the TES (eg, content, technology delivery, and support) and not a failure of the individual using the program. It is also important that supporters never assume that a failure is related to a specific aspect of the consumer’s identity. Rather, cultural responsivity is one possible lens through which to examine challenges with program use, fit, and skill acquisition and application.

#### Usability

Usability refers to the ease of use of a technology and relates to the time, effort, or capabilities required to use a TES. Usability testing is needed in both research and real-world settings to identify technical issues (eg, bugs) and nontechnical issues (eg, poor flow or consumer experience) and to make the technology as intuitive as possible. Usability is most often evaluated using self-report measures, which best maps on to perceived usability, but determining the actual usability of a TES requires observing people’s use of it often in these research or real-world settings. Cultures or identities might impact what technologies are in fact usable for specific consumers. In fact, significant usability barriers have been reported with commercially available apps in a sample of older and lower-income individuals who were also racially diverse, related to low confidence with using technology and frustration with certain design features [30]. Existing research indicates that there are differences in digital literacy across age, race, income, and education level [71-73]. For example, older adults access the internet less frequently and, on average, report fewer internet- and computer-based skills than younger adults, and older adults with higher income and education report higher internet access and use than their peers [71]. However, older adults who are Black or Latinx are less likely to use technology for health-related purposes than their White peers [72]. In response, support should include an orientation to the technology, perhaps through a brief initial session, to go over how to navigate the program and address any questions or challenges [74,75]. Support interactions can be used to troubleshoot usability issues, provide individuals’ options to tailor the TES (eg, select text size, select the type of tracking log, and select notification preferences), and acknowledge the potential limitations of a specific TES. Depending on the context of the support interaction (ie, research vs clinical setting), the supporter may also be able to recommend a different TES that may be more acceptable if usability barriers persist.

#### Engagement

Engagement refers to a state related to use of a technology and consists of behavioral, cognitive, and affective components [76]. Individuals may engage with programs differently, and the factors that impact engagement might differ across individuals and be shaped by culture. Some individuals may engage with a program for shorter periods more frequently, whereas others may engage for longer periods less often. Research has identified different patterns of engagement that were related to outcomes across a CBT-based TES in a large sample of adults with depression and anxiety [77]. Additional research is needed to examine how engagement may differ in various populations. In addition to baseline differences in engagement, individuals may have experiences using a TES that may promote disengagement, including another type of failure (eg, usability and fit) that then leads to an engagement failure. Ultimately, lower engagement may lead to a lack of benefit from a program, as previous research has found that higher engagement with a TES is linked to better outcomes [77]. Therefore, engagement is an important variable for supporters to assess and respond to.

#### Fit

Fit refers to how well a program meets an individual’s needs and symptoms. A recent systematic review found that perceived fit of a TES was a facilitator of engagement, highlighting the importance of attending to fit [33]. On the basis of the Efficiency Model, a fit failure occurs when an individual feels that a program is not a good match for them, perhaps because the program does not seem to be designed for someone with their identities or experiences. This could lead to engagement and other failures. For example, someone may notice that the people in the examples do not look like them or that relevant experiences (eg, systemic barriers) or aspects of their identities (eg, religion or spirituality) or values (eg, collectivism, community engagement, and family) are not represented. If an individual reports concerns about the fit of a TES, the supporter can attempt to address their concerns, validate their experience, and tailor the program to them, which may in turn lead to the individual engaging with the program. People may not always want to discuss personal experiences, concerns, or experiences of marginalization with supporters. In this case, the supporter should respect the individual’s preferences. However, if an individual does want to discuss these experiences or raise questions with the supporter or if they feel it is relevant to their symptoms or goals, the supporter needs to be trained to respond appropriately.

If individuals do not feel that the interventions are designed for them and support does not address these concerns and potential problems or a supporter responds in a harmful or invalidating way, they may understandably discontinue use of the TES. Furthermore, the intervention may not be helpful to that individual, and in a worst-case scenario, it may be harmful. It is important to note that a specific TES may not be a good fit for some individuals, and the best outcome may be for them to consider another TES or treatment that may be a better fit.

#### Knowledge

Knowledge refers to whether someone is able to gain the relevant knowledge from a TES. This might be knowledge gained from psychoeducational content or skill development through experiential tools or lessons. Knowledge failures occur when an individual does not extract what the developers intended to teach through a TES, for example, not learning the difference between an emotion and thought in a digital CBT product or the signs and symptoms of anxiety in material intended to teach that. The lack of cultural responsivity might result in knowledge failures when TESs are not designed in ways that express cultural differences. For example, psychoeducational content focused on teaching about the signs, symptoms, and prevalence of anxiety may fail to include different symptom presentations across cultures or prevalence rates in specific subpopulations. When thinking about specific behavior change strategies or skills, knowledge includes how to use a skill or tool flexibly, based on an individual’s culture, context, values, and experiences. Therefore, a knowledge failure could also occur when a specific behavior change strategy is not represented in a culturally responsive way within a TES. For example, a program may include assertiveness training without providing additional information regarding when this skill may or may not be helpful or consistent with an individual’s culture or values.

Supporters can address these knowledge gaps by correcting or supplementing the TES with culturally specific examples or tailoring. For example, a supporter could supplement the introduction of an assertiveness training skill by asking how an individual wants to interact with their family members, friends, colleagues, and communities and to help them find a balance that respects their values (eg, connection, family, and independence) without the supporter imposing their own values on the individual. Supporters also need to be able to assist people in using skills in a way that is culturally responsive and helpful in general. For example, if an individual restructures a thought related to an experience of discrimination, it is important that the supporter responds to validate the individual’s experience, acknowledge systemic inequities, and provide additional information about how the skill could be used in a culturally responsive way (eg, restructuring internalized thoughts related to experiences of discrimination). This way the individual has a full understanding of the skill and how they can apply it in a manner that will hopefully be helpful to them.

#### Implementation

Implementation failures, where people do not apply skills in their daily lives, may occur for several reasons and may be related to a lack of cultural responsivity. For example, an individual may have acquired the ability to use the skill of scheduling pleasant activities (ie, they have the knowledge) but may not apply the skill in their life. It is possible that the skill was presented in a way that does not seem to fit with the individual’s context (eg, examples of specific activities were not accessible due to cost or other barriers). If the supporter asks about barriers to implementation, they may be able to gain a better understanding of the individual’s context and may be able to provide more helpful suggestions to address relevant barriers.

#### Additional Support Recommendations

Published consensus on training standards for supporters is lacking [78]. Training can be delivered via workshops and then followed by regular supervision [78,79]. Existing trainings often include background information on TESs, a review of the empirical evidence for these approaches, and a discussion of professional issues, including ways to communicate effectively via written communication [78]. We suggest that these trainings should also include a focus on cultural responsivity, so that supporters are able to provide ethical and quality support. It is critical that supporters consider their own identities, the identities of the individuals they will work with, and how these identities may impact people’s experiences without making assumptions. Existing exercises and frameworks of cultural responsivity, such as the “ADDRESSING” framework [35], which facilitates recognition and consideration of a range of identities and intersectionality, could be used to facilitate this type of awareness in trainings. Supporters also need to be able to acknowledge systemic inequities and experiences of discrimination in our society, understand how to apply the specific skills within a TES in a way that is culturally responsive, and be able to have open written and phone-based communication about these topics. Therefore, trainings should also include information about systemic inequities and discrimination. Existing training material and resources on cultural responsiveness and cultural humility for face-to-face interventions can be adapted for supporter training [35,40,42,44,80]. Ideally, these trainings should also offer opportunities to practice using these skills before providing support (eg, responding to sample messages to apply the knowledge learned with ongoing supervision). Cultural responsivity could then be added to existing measures of supporter fidelity for internet-delivered CBT and assessed [81]. This could help to increase the cultural responsiveness of human support in several ways. First, these fidelity checklists could be used to train new supporters. Although cultural responsiveness requires a lifelong commitment and is not something that can be fully achieved, it could be beneficial to require the demonstration of a certain level of cultural responsiveness as part of the initial training. These checklists could also be used to monitor ongoing support messages longitudinally. In addition, including cultural responsiveness in a fidelity checklist centers it and ensures that it is formally part of what is required of supporters. Finally, having a concrete way to assess this construct in support would allow for more research into the relations among cultural responsiveness, engagement, and outcomes in TESs, with the ultimate goal of providing high-quality responsive support for all.

We also recommend including human-centered design and crowdsourcing methods in the development of support protocols. For example, 1 recent innovative study used human-centered design, crowdsourcing, and researcher expertise to develop SMS text messages for a smartphone app for low-income individuals [82]. These methods could be used to develop other messages sent to individuals using TESs, including support protocols.

As previously noted, support, including the frequency (ie, weekly or at a set interval [push] or as requested by the user [pull]) and amount (ie, brief SMS text messages, longer written messages, or phone calls) of support, varies across TESs. Therefore, cultural responsivity will need to be considered within the context of each TES and its support protocol. We present considerations for delivering culturally responsive human support in Multimedia Appendix 1. Some of these examples include attempts to increase cultural responsivity through supporter training and in initial contact, whereas others focus on cultural responsivity throughout the dynamic process of the individual using the TES and interacting with the supporter.

## Conclusions and Future Directions

We provide a broad framework for considering cultural responsivity in TESs, including targets for adapting, tailoring, or designing TESs at the level of program content and support, based on existing models. We provide specific recommendations for how content and support can address each aspect of cultural responsivity by (1) demonstrating awareness of the significance of differing identities, (2) demonstrating knowledge about different experiences related to identities, and (3) tailoring content to a unique individual. In terms of content, we suggest that the BIT Model can be used through a lens of cultural responsivity to consider specific targets for adaptations, tailoring, and design across both technical and clinical components of programs. With regard to support, we suggest that a lens of cultural responsivity can be used in combination with the Efficiency Model of Support to consider how the lack of cultural responsivity may contribute to common problems within TESs (ie, usability, engagement, fit, knowledge, and implementation failures) and to then use support to increase cultural responsivity and acknowledge and respond to these problems. Furthermore, we recommend that supporter training include an emphasis on cultural responsivity. The use of this framework and recommendations could promote further research on cultural responsivity in TESs, additional training methods for supporters, and subsequent efforts to examine the positive impact this may have on individuals’ experiences, engagement with TESs, and outcomes. Ultimately, these recommendations could increase the cultural responsivity of TESs, thereby increasing access to effective, ethical, and responsive interventions and in turn reducing mental health treatment disparities.

Space limitations and our desire to present a flexible, broad, usable framework preclude us from providing exhaustive, inclusive examples throughout. We also drew from specific areas of expertise of the authors, including a focus on examples addressing systemic inequities and discrimination. We hope this paper will spur additional work in this area and that other authors, researchers, and clinicians will use their expertise in various aspects of cultural responsivity (eg, religion and spirituality, language and translation, and different beliefs about what it means to be “healthy”) to expand on this work. In addition, to move the field forward, we recommend that researchers measure a broad range of identities in their samples so that this information can be reported and additional questions regarding effectiveness, engagement, and use can be examined. For example, in a recent literature review, Nouri et al [29] found that only a small number of studies evaluated program use by individual characteristics and that those that did, only reported one to several characteristics, most commonly age and gender.

In conclusion, cultural responsivity should be considered at the level of both program content and human support in TESs. Existing frameworks related to TESs and foundational work on cultural responsivity in face-to-face interventions can be used to advance cultural responsivity in TESs. This work is critical to address the mental health treatment disparities seen in face-to-face treatments and TESs to date and for TESs to reach their full potential to increase access to effective and inclusive mental health treatment.

## Appendix

Multimedia Appendix 1 Examples of culturally responsive human support.

## Abbreviations

BIT: Behavioral Intervention Technology
CBT: cognitive behavioral therapy
TES: technology-enabled service
