# Supplementary material for: Cultural Responsivity in Technology-Enabled Services: Integrating Culture Into Technology and Service Components
Source: J Med Internet Res. 2023 Oct 3;25:e45409. doi: 10.2196/45409 (PMC10582817; doi:10.2196/45409)
Supplement: Multimedia Appendix 1 [file jmir_v25i1e45409_app1.docx]

**Multimedia Appendix 1.** Examples of culturally responsive human support.

| Type of consideration and situation or content | | | | How the failure is identified | Recommendation for supporter response or training | Aspects of cultural responsivity ^a^ |
| --- | --- | --- | --- | --- | --- | --- |
| **Supporter training** | | | | | | |
|  | Standard training | | | Standard procedure | Supporters complete cultural responsivity training so they can consider the domains of identity for individuals and themselves, acknowledge systemic inequities, validate experiences of discrimination, and help individuals tailor skills to their unique experiences. | (1), (2), and (3) |
|  | Start of program | | | Standard procedure | In the initial message, the supporter asks each person for information about themselves and important aspects of identity. Note that the supporter is there to help them apply program content to their goals and contexts. State that the supporter is there for any questions or concerns about the program to open the door for future communication. | (1), (2), and (3) |
| **Failures from the Efficiency Model^b^** | | | | | | |
|  | **Usability** | | | | |  |
|  |  | | Technology is less familiar and/or usable for some individuals | Standard procedure; an individual reports a usability problem or does not use the program in a set period | Initial orientation to the program and technology. Ask about challenges with technical aspects of the program. Assist the individual in using the technology, offer support, and provide options to tailor the technology (eg, options around notifications, font size, and types of tracking logs). | (3) |
|  | **Engagement** | | | | |  |
|  |  | | The individual does not access the program | The individual reports a challenge with engagement or has not accessed the program for a set period | Consider that there may be different engagement styles. The supporter asks about concerns about the program, challenges with motivation, and barriers to engagement. The supporter may encourage the individual to revisit their goals, schedule a specific time or set reminders to use the program, or offer additional support contact. | (3) |
|  | **Fit** | | | | |  |
|  |  | | A lack of representation in pictures, names, and examples | The individual raises concerns to a supporter. | Acknowledge differing identities and experiences. The supporter acknowledges this program limitation, states they are there to help the individual think about how the content could be helpful to them in their life, and offer to discuss further. Feedback should be shared with developers as well to inform product improvements. | (1), (2), and (3) |
|  | **Knowledge and implementation** | | | | |  |
|  |  | | Psychoeducation, a cycle of avoidance | The individual asks a question about avoidance in the context of discrimination. | Acknowledge systemic inequities in the society, validate the experience, and review psychoeducation that avoidance is adaptive in certain contexts and that avoiding discrimination (when possible) can be adaptive and is a personal decision. Note that it can be helpful to intentionally make this decision to avoid versus avoiding on autopilot and offer additional discussion if it would be helpful. | (1), (2), and (3) |
|  |  | | Cognitive restructuring | The individual asks whether this skill applies to thoughts about the validity of an experience of discrimination or the supporter notices the skill has been used in this way in the program. | Acknowledge and validate the experience of discrimination. Clarify that we do not use cognitive restructuring to question the validity of experiences (eg, whether something was racist, sexist, homophobic, classist, or ableist). Validate the experience of discrimination. Ask the individual to consider if the experiences of discrimination are related to any internalized thoughts about themselves (eg, I am not worthy) and encourage the individual to reframe or think more flexibly about those internalized thoughts. | (1), (2), and (3) |
|  |  | | Behavior change | The individual asks a question related to behavior change that includes culture or an experience with discrimination | Encourage the individual to use their goals and values to guide behavior change and exposures and to consider context. State that it is OK to make different decisions in different contexts based on goals and values, and these decisions may relate to culture, worldview, or experiences with discrimination. For example, one may or may not make a different decision about how to respond to a problematic comment made at work versus in another context based on their values and goals. Or one may decide that they want to practice being more assertive in work situations but not with family members, if that is consistent with their values. | (1), (2), and (3) |

^a^(1) Demonstrating awareness of the significance of differing identities; (2) acquiring and demonstrating knowledge about different experiences related to identities; (3) tailoring goals and practices to the unique values and life experiences of the individual [36].

^b^Efficiency Model=Efficiency Model of Support.
